# Supplementary material for: Multivessel versus Culprit-Only Percutaneous Coronary Intervention in Patients with Non-ST-Elevation Acute Coronary Syndrome
Source: J Clin Med. 2022 Oct 18;11(20):6144. doi: 10.3390/jcm11206144 (PMC9605400; doi:10.3390/jcm11206144)
Supplement: Supplementary file 1 [file jcm-11-06144-s001.zip › jcm-1966596-supplementary.pdf]

Supplemental Materials

The following physicians are members of the PCI Registration Committee of the NHR. They represent the hospitals that have provided the PCI data for this study.

M	Scholte	Albert Schweitzer Hospital
M	Meuwissen	Amphia
JP	Henriques	Amsterdam University Medical Centre, University of Amsterdam
KMJ	Marques	Amsterdam University Medical Centre, VU Medical Centre
T	Teeuwen	Catharina Hospital
H	Al Hashimi	Canisius Wilhelmina Hospital
M	Magro	Elisabeth-TweeSteden Hospital
J	Daemen	Erasmus Medical Centre
BJ	Sorgdrager	Haaglanden Medical Centre
CE	Schotborgh	Haga Hospital
V	Roolvink	Isala
J	Polad	Jeroen Bosch Hospital
R	Scherptong	Leiden University Medical Centre
M	Van der Ent	Maasstad Hospital
AWJ	Van 't Hof	Maastricht University Medical Centre
F	Spano	Meander Medical Centre
J	Brouwer	Medical Centre Leeuwarden
MG	Stoel	Medical Spectrum Twente
A	Dedic	Noordwest Hospital Group
G	Amoroso	OLVG
C	Camaro	Radboud University Medical Centre
PW	Danse	Rijnstate
JP	Van Kuijk	St. Antonius Hospital
EK	Arkenbout	Tergooi
WT	Ruifrok	Treant Zorggroep, Scheper Hospital
A	Kraaijeveld	University Medical Centre of Utrecht
E	Lipsic	University Medical Centre Groningen
S	Aydin	VieCuri Medical Centre
R	Erdem	ZorgSaam Hospital
AWJ	Van 't Hof	Zuyderland Medical Centre

The following physicians are members of the Cardiothoracic Surgery Registration Committee of the NHR. They represent the hospitals that have provided the CABG data for this study.

S	Bramer	Amphia
WJP	Van Boven	Amsterdam University Medical Centre, University of Amsterdam
ABA	Vonk	Amsterdam University Medical Centre, VU Medical Centre
BMJA	Koene	Catharina Hospital
JA	Bekkers	Erasmus Medical Centre
GJF	Hoohenkerk	Haga Hospital
ALP	Markou	Isala
A	De Weger	Leiden University Medical Centre
P	Segers	Maastricht University Medical Centre
F	Porta	Medical Centre Leeuwarden
RGH	Speekenbrink	Medical Spectrum Twente
W	Stooker	OLVG
WWL	Li	Radboud University Medical Centre
EJ	Daeter	St. Antonius Hospital
NP	Van der Kaaij	University Medical Centre of Utrecht
YL	Douglas	University Medical Centre Groningen

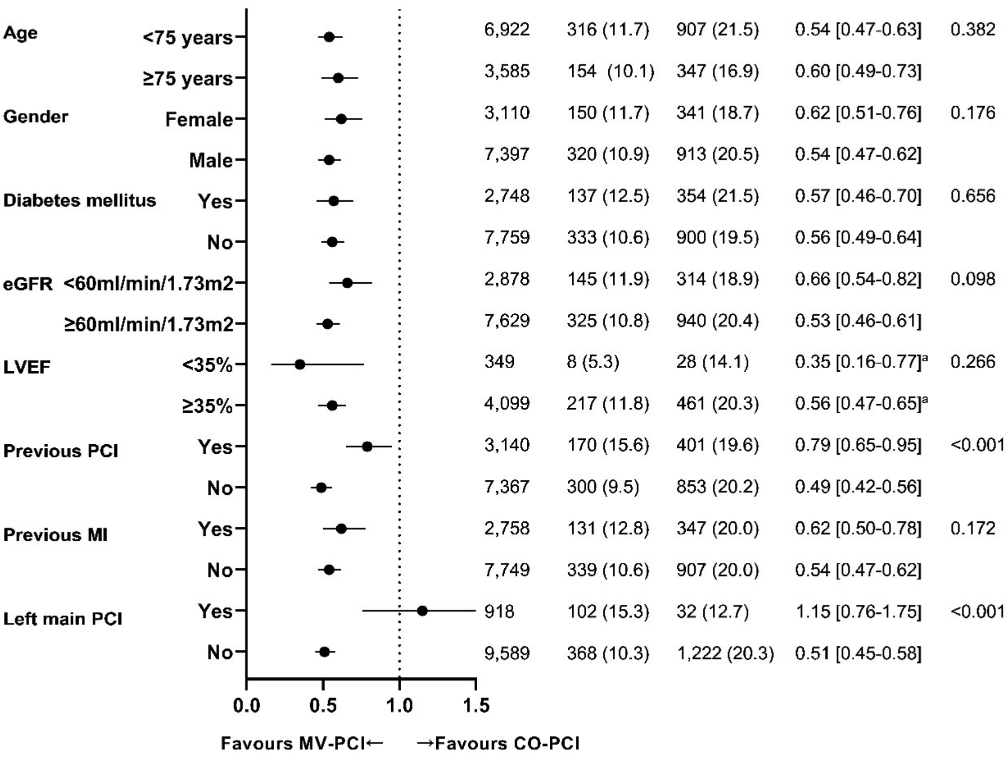

Subgroup		N cases	N reinterventions (%) MV-PCI	N reinterventions (%) CO-PCI	Adjusted HR* [95% CI]	Interaction p-value
Age	<75 years	6,922	316 (11.7)	907 (21.5)	0.54 [0.47-0.63]	0.382
	≥75 years	3,585	154 (10.1)	347 (16.9)	0.60 [0.49-0.73]	
Gender	Female	3,110	150 (11.7)	341 (18.7)	0.62 [0.51-0.76]	0.176
	Male	7,397	320 (10.9)	913 (20.5)	0.54 [0.47-0.62]	
Diabetes mellitus	Yes	2,748	137 (12.5)	354 (21.5)	0.57 [0.46-0.70]	0.656
	No	7,759	333 (10.6)	900 (19.5)	0.56 [0.49-0.64]	
eGFR	<60ml/min/1.73m2	2,878	145 (11.9)	314 (18.9)	0.66 [0.54-0.82]	0.098
	≥60ml/min/1.73m2	7,629	325 (10.8)	940 (20.4)	0.53 [0.46-0.61]	
LVEF	<35%	349	8 (5.3)	28 (14.1)	0.35 [0.16-0.77][a]	0.266
	≥35%	4,099	217 (11.8)	461 (20.3)	0.56 [0.47-0.65][a]	
Previous PCI	Yes	3,140	170 (15.6)	401 (19.6)	0.79 [0.65-0.95]	<0.001
	No	7,367	300 (9.5)	853 (20.2)	0.49 [0.42-0.56]	
Previous MI	Yes	2,758	131 (12.8)	347 (20.0)	0.62 [0.50-0.78]	0.172
	No	7,749	339 (10.6)	907 (20.0)	0.54 [0.47-0.62]	
Left main PCI	Yes	918	102 (15.3)	32 (12.7)	1.15 [0.76-1.75]	<0.001
	No	9,589	368 (10.3)	1,222 (20.3)	0.51 [0.45-0.58]	

Figure S1. Subgroup analysis on reinterventions between MV-PCI and CO-PCI (elective revascularisations <6 weeks excluded). Values are *n* (%) or HR (95% CI). CI, confidence interval; CO, culprit-only; eGFR, estimated glomerular filtration rate; HR, hazard ratio; MI, myocardial infarction; MV, multivessel; LVEF, left ventricular ejection fraction; PCI, percutaneous coronary intervention. * Covariates used for correction: age, gender, diabetes mellitus, dialysis, vessel treatment and multivessel PCI. [a] Data depicted represent unadjusted HR due to missing data assumed to be not missing at random.

Table S1. Baseline characteristics with multiple imputed data.

	Overall cohort			
	MV-PCI N = 4,235	CO-PCI N = 6,272	OR [95%-CI]	p-value
**Age**	69.1 ±11.6	68.4 ±11.4	NA	0.004
**Gender, female**	1,286 (30.4)	1,824 (29.1)	1.06 [0.98-1.16]	0.157
**eGFR <60ml/min/1.73m2**	1,220 (28.8)	1,659 (26.5)	0.89 [0.80-0.98]	0.023
**Diabetes mellitus**	1,100 (26.0)	1,648 (26.3)	0.98 [0.90-1.08]	0.730
**LVEF <35%**	151 (7.6)	198 (8.0)	1.06 [0.85-1.33]	0.588
**Dialysis**	37 (1.0)	51 (0.9)	1.09 [0.71-1.67]	0.683
**Previous MI**	1,027 (24.2)	1,731 (27.6)	0.84 [0.77-0.92]	<0.001
**Previous PCI**	1,093 (25.8)	2,047 (32.6)	0.72 [0.66-0.78]	<0.001
**Radial approach**	3,555 (83.9)	5,298 (84.5)	0.96 [0.80-1.14]	0.589
**Left main PCI**	666 (15.7)	252 (4.0)	4.46 [3.83-5.18]	<0.001
**Treated vessels**<br>**LAD**<br>**RCX**<br>**RCA**<br>**LAD + RCX**<br>**LAD + RCA**<br>**RCX + RCA**<br>**LAD + RCX + RCA**	2,081 (49.1)<br>1,106 (26.1)<br>630 (14.9)<br>416 (9.8)	2,537 (40.4)<br>1,562 (24.9)<br>1,904 (30.4)	-*	-*
**Lesions treated**	2 [2-3]	1 [1-1]	NA	<0.001
**Stent treatment**<br>**DES**<br>**BMS**<br>**BRS**<br>**Unknown**	3,501 (84.4)<br>6 (0.1)<br>0 (-)<br>643 (15.5)	5,083 (88.9)<br>19 (0.3)<br>6 (0.1)<br>611 (10.7)	4.72 [3.74-5.95]	<0.001
**Balloon dilatation**	365 (8.6)	525 (8.4)	1.03 [0.90-1.19]	0.654
**Other treatment**	174 (4.1)	203 (3.2)	1.28 [1.04-158]	0.019

Values are mean ±standard deviation, *n* (%) or median (interquartile range). BMS, bare metal stent; BRS, bioresorbable vascular scaffold; DES, drug eluting stent; CO-PCI, culprit-only PCI; eGFR, estimated glomerular filtration rate; MI, myocardial infarction; MV-PCI, multivessel PCI; NA, not applicable; OR, odds ratio; LVEF, left ventricular ejection fraction; PCI, percutaneous coronary intervention. * p-value could not be determined since there was no comparator.

Table S2. Cox proportional model of multivariable analysis of predictors of reinterventions at long-term follow-up.

	**Univariable**	**Multivariable ***
**Age**	0.99 [0.99-1.00]	0.99 [0.99-1.00]
**Gender, female**	0.95 [0.86-1.06]	1.02 [0.91-1.14]
**eGFR <60ml/min/1.73m2**	1.00 [0.90-1.13]	NA
**Diabetes mellitus**	1.16 [1.04-1.29]	1.12 [1.00-1.26]
**LVEF**	0.66 [0.47-0.92]	NA
**Dialysis**	2.06 [1.38-3.05]	2.08 [1.40-3.09]
**Previous MI**	1.10 [0.99-1.22]	1.00 [0.87-1.14]
**Previous PCI**	1.16 [1.05-1.29]	1.07 [0.94-1.21]
**Radial approach**	0.95 [0.82-1.09]	NA
**Left main PCI**	0.91 [0.77-1.09]	NA
**Vessel treatment**		
**Stent treatment**	0.47 [0.40-0.55]	0.66 [0.55-0.79]
**Balloon dilatation**	1.41 [1.21-1.64]	NA
**Other treatment**	1.91 [1.56-2.34]	1.65 [1.31-2.09]
**Multivessel PCI**	0.53 [0.47-0.59]	0.56 [0.50-0.63]

Values are hazard ratio (95% confidence interval). eGFR, estimated glomerular filtration rate; LVEF, left ventricular ejection fraction; MI, myocardial infarction; NA, not applicable; PCI, percutaneous coronary intervention. * Covariates used for correction: age, gender, diabetes mellitus, dialysis, previous MI, previous PCI, stent treatment, other treatment and multivessel PCI.
